# The impact of applying various de novo assembly and correction tools on the identification of genome characterization, drug resistance, and virulence factors of clinical isolates using ONT sequencing

**DOI:** 10.1186/s12896-023-00797-3

**Published:** 2023-07-31

**Authors:** Hussain A. Safar, Fatemah Alatar, Kother Nasser, Rehab Al-Ajmi, Wadha Alfouzan, Abu Salim Mustafa

**Affiliations:** 1 OMICS Research Unit, Health Science Centre, Kuwait University, Hawalli Governorate, Kuwait; 2 Serology and Molecular Microbiology Reference Laboratory, Mubarak Al-Kabeer Hospital, Ministry of Health, Hawalli Governorate, Kuwait; 3 Department of Microbiology, Faculty of Medicine, Kuwait University, Hawalli Governorate, Kuwait; 4 Microbiology Unit, Farwaniya Hospital, Ministry of Health, Al Farwaniyah Governorate, Kuwait

**Keywords:** Genome assembly, ONT, *E. coli*, WGS

## Abstract

Oxford Nanopore sequencing technology (ONT) is currently widely used due to its affordability, simplicity, and reliability. Despite the advantage ONT has over next-generation sequencing in detecting resistance genes in mobile genetic elements, its relatively high error rate (10–15%) is still a deterrent. Several bioinformatic tools are freely available for raw data processing and obtaining complete and more accurate genome assemblies. In this study, we evaluated the impact of using mix-and-matched read assembly (Flye, Canu, Wtdbg2, and NECAT) and read correction (Medaka, NextPolish, and Racon) tools in generating complete and accurate genome assemblies, and downstream genomic analysis of nine clinical *Escherichia coli* isolates. Flye and Canu assemblers were the most robust in genome assembly, and Medaka and Racon correction tools significantly improved assembly parameters. Flye functioned well in pan-genome analysis, while Medaka increased the number of core genes detected. Flye, Canu, and NECAT assembler functioned well in detecting antimicrobial resistance genes (AMR), while Wtdbg2 required correction tools for better detection. Flye was the best assembler for detecting and locating both virulence and AMR genes (i.e., chromosomal vs. plasmid). This study provides insight into the performance of several read assembly and read correction tools for analyzing ONT sequencing reads for clinical isolates.

**Supplementary Information:**

The online version contains supplementary material available at 10.1186/s12896-023-00797-3.

## Introduction

The rapid development of whole-genome sequencing tools expanded their usage to sequence the whole genome of species from small single cells to large and complex species [1]. Besides, the reduction in sequencing costs and simplicity in library preparations allowed a worldwide distribution of these sequencing tools, where low- and mid-income countries have great access to such advanced tools [2, 3]. Next-generation sequencing (NGS) technology has wholly revolutionized genome analysis. NGS is relatively timesaving in that it can promptly verify a sample sequence type and the presence of critical genes [4, 5]. The production of millions of short reads (mostly < 150 bp in length) during NGS and a low error rate (< 2%) makes this technology highly accurate and reliable in identifying single nucleotide polymorphisms (SNPs) and understanding population structures [6]. Despite the advantages of using short reads, especially with sequencing accuracy, the major shortcomings of using short reads are the failure to assemble complete genomic structures and poor gene organization even with special library preparations that intend to increase the depth of sequencing coverage, and the inability to resolve all genomic repeats [7, 8]. The location of resistance genes is critical epidemiologically -especially for public health laboratories- and unresolved/incomplete assembled genomes cannot indicate whether the resistant gene is located on the chromosome or the mobile genetic elements (MGEs) [9, 10]. Another drawback of NGS is the possible introduction of a biased nucleotide variance during the PCR amplification step of library preparation [11].

To conquer the hindrance of unresolved assemblies and inaccurate repetitive region sequencing, Oxford Nanopore Sequencing Technology (ONT), a third-generation sequencing technology, generates long reads that exceed the length of repeated regions resulting in complete genome assemblies with more accurate gene locations (i.e., chromosomal vs. MGEs) [9, 12]. ONT generates reads ranging from 500 bp to 2.3 Mb, with 10–30 kb genomic libraries being the most prevalent [13]. The main limitation of ONT is the relatively higher error rate (~ 10–15%) compared to NGS, though the technology is constantly improving [14]. Computational efforts were made to develop assembly and correction tools to transform raw ONT signals into completely assembled genomes, taking into consideration both the high error rates and the length of reads. The assembly algorithms and correction tools aim to reduce the high error rates.

Several long-read assemblers are freely available online. These assemblers use different algorithms to generate the best consensus sequences in combination with a secondary correction tool. Flye is a long-read de novo assembler based on a generalized Bruijn Graph. Flye combines multiple disjoint genomic segments, connects all error-prone disjointigs into a single string, and outputs an accurate assembly graph [15]. Flye package can be called a ‘complete pipeline’ as this tool converts raw ONT reads into corrected consensus sequences [16]. Canu is an assembler based on the over-layout-consensus algorithm (OLC) designed for reconstructing noisy long reads [17]. Canu operates in three phases: correction, trimming, and assembly. Canu improves base calling error in the correction phase by trimming low-quality bases and adapters in the trimming phase, and finally arranges the reads into contigs to generate both consensus sequences and graphs of alternate paths in the assembly phase [17, 18]. Wtdbg2 is another assembler that is based on the OLC algorithm. Wtdbg2 cleaves long reads into 1024 segments, merges similar segments into a vertex, and connects vertices based on segment adjacency on reads producing a fuzzy de Bruijn graph [19]. A major advantage of using Wtdbg2 is its speed of assembly, which can be 10–17 times faster than other assemblers such as Canu to produce comparable consensus sequences. However, Wtdbg2 assembles raw reads without an error correction step [19]. NECAT is a novel two-stage assembler for noisy long-reads designed to overcome complex errors in Nanopore reads. NECAT corrects and assembles raw reads into high-quality consensus sequences relatively faster than Canu [20]. To generate genome assemblies with a reduced error rate, read correction tools -or polishing tools- are often required. Though the correction step takes longer than genome assembly, the ‘corrected/polished’ assembly is more complete and is much improved [21]. For example, NextPolish contains two core modules and uses a stepwise mode to fix base errors (SNV/Indel) [22], while Racon is intended as a standalone consensus module to correct contigs generated by assembly methods [23].

The evaluation and benchmarking of various long-read assembly tools using reference laboratory strains have been widely explored in the literature [11, 18, 24]. However, benchmarking the usage of assembly and read correction tools in clinical isolates has not been sufficiently investigated. In this study, we assessed and compared the capability of mix-and-matched assembly and read correction tools in generating complete and accurate genome assemblies, and downstream genomic analysis regarding strain and serotype identification, annotation, detection of antimicrobial resistance genes, plasmid finding, and virulence potential of nine clinical *Escherichia coli* isolates.

## Materials and methods

The sequencing reads were submitted to EMBL's European Bioinformatics Institute and are available online with accession numbers ERR10468513- ERR10468521 (Suppl. S1 Table S1) available at: https://www.ebi.ac.uk/ena/browser/view/PRJEB57325.

### Bacterial isolates, library preparation, MinION sequencing, and reads preparation

*E. coli* isolates (*n* = 9) were obtained from rectal swabs of pregnant women admitted to the Gynecology ward in Farwaniya Hospital, Kuwait. All methods and ethical approvals were obtained and performed in accordance with the Ethical Committees of the Health Sciences Centre, Kuwait University, and the Ministry of Health, Kuwait. The patients/participants (or their legal guardians) provided their written informed consent to participate in this study. These bacterial isolates were grown overnight at 37 °C on selective agar, and single colonies were suspended in phosphate-buffered saline (PBS). Bacterial genomic DNA was purified using Monarch genomic DNA purification kit (New England BioLabs) as follows. Bacterial cell pellets from PBS were generated by centrifugation at 10,000 g for 5 min. The pellet was resuspended with 1 µl of proteinase K and 3 µl of RNAse, and then 100 µl of cell lysis buffer was added. The samples were vortexed and incubated for 5 min at 56 °C with agitation. Genomic DNA was extracted from the lysed samples following Part 2 of the kit protocol for cultured cells from the binding step onwards. The extracted DNA was checked for quality and quantity using a spectrophotometer (Nanodrop, ThermoFisher Scientific), and a fluorometer (Qubit, ThermoFisher Scientific), respectively.

Oxford Nanopore Ligation Sequencing kit (SQK-LSK109) with Native Barcoding Expansion 1–12 (EXP-NBD104) was used for library preparation. Following the kits protocols, 1 µg of the isolated genomic DNA was treated with end-repair/dA tailing module, and then DNA was eluted after AMPure XP bead clean up. The genomic DNA was barcoded following the Native Barcoding Expansion 1–12 (EXP-NBD104) protocol and then cleaned with AMPure XP beads. The eluted barcoded genomic DNA was pooled to 65 µl and used for adapter ligation. The final library contained 29.8 ng DNA and 50 fmol of the library was loaded onto an R9.4 flow cell. The sequencing was performed using the MiniON Mk1C device with a flow cell (FLO-MI106D) – containing 875 available pores) following the user manual (Suppl. S1 Table S2). The run proceeded for the full 48 h.

The built-in Guppy v4.0.11 (https://community.nanoporetech.com) base-called and demultiplexed the fast5 reads and output fastq files. Barcodes and adapter sequences were then trimmed from reads using Porechop (v0.2.1, https://github.com/rrwick/Porechop). The resulting 9 demultiplexed, barcode-free read sets were deposited under accession numbers ERR10468513- ERR10468521 (Suppl. S1 Table S1). NanoPlot and Nanofilt were used to assess the reads quality and filter reads quality to gather reads with q score > 8 (Suppl. S1 Table S3) [25].

### De novo assembly, read correction, and assembly assessment

Four de novo genome assemblers: Flye (version 2.8.3-b1695), Canu (version 1.9), Wtdbg2 (version 3.0), and NECAT (version 0.0.1), and three read correction tools: Medaka (version 0.11.0), NextPolish (version 1.4.1), and Racon (version 1.4.10) were selected for assembling and correcting long reads generated by ONT [15, 17, 19, 20, 22, 23, 26]. The reads for each isolate were assembled using each of above mentioned de novo assemblers with default settings. After each assembly, the consensus files generated followed one round of read correction using Medaka, NextPolish or Racon with default settings. The running time and CPU usage are available in Suppl. S1 Table S4. This resulted in generating 12 corrected assemblies per read set. The consensus sequences generated from each corrected assembly underwent a similarity sequence against the nucleotide database NCBI Basic Local Alignment Search Tool using BLAST + Command Line Application tool v.2.12.0 for large contigs (> 1,000,000 bp) [27]. The *E. coli* serotype determination was performed using ECTyper (version 1.0.0) [28]. The quality of each corrected assembly was assessed using QUAST (version 5.0.2) [29] –using the LG parameter- comparing them with appropriate reference genome for each isolate (based on BLAST + results). The total length (bp), number of contigs, GC% and total aligned sequence (bp) were evaluated in each corrected assembly.

### Identification of genes annotation, antimicrobial resistance genes, plasmids, and virulence genes

The *E. coli* genomes were annotated using Prokka (version 1.14.5) [30] and the generated GFF files were used as input for pangenome inference using Roary (version 3.13.0) [31] to generate the core- (genes present in all analyzed isolates), shell- (genes present in the majority of genomes), and cloud- (genes present in the minority of the genomes) genes. Antimicrobial resistance genes were detected using staramr (version 0.7.2) [32] against known gene sequences in the ResFinder database [33] with 98% minimum identity and 60% minimum coverage and using Resistance Gene Identifier (RGI) strict criteria [34]. Plasmids were identified using staramr against known plasmid sequences in the PlasmidFinder database [35] with 98% minimum identity and 60% minimum coverage. Virulence genes were identified using ABRicate (version 2.0) integrated with Virulence Factors Database (VFDB) with 98% minimum identity and 60% minimum coverage [36, 37], and Venn diagrams were constructed using online tool https://bioinformatics.psb.ugent.be/webtools/Venn/.

### Statistical analysis

Wilcoxon signed-rank test was performed using GraphPad Prism (California, USA) (version 9.4.1) to determine whether significant differences (*p* < 0.05) existed between assembled genomes with and without using read correction tools in total length (bp), GC%, total aligned (bp), and indels.

## Results and discussion

**Fig. 1:**
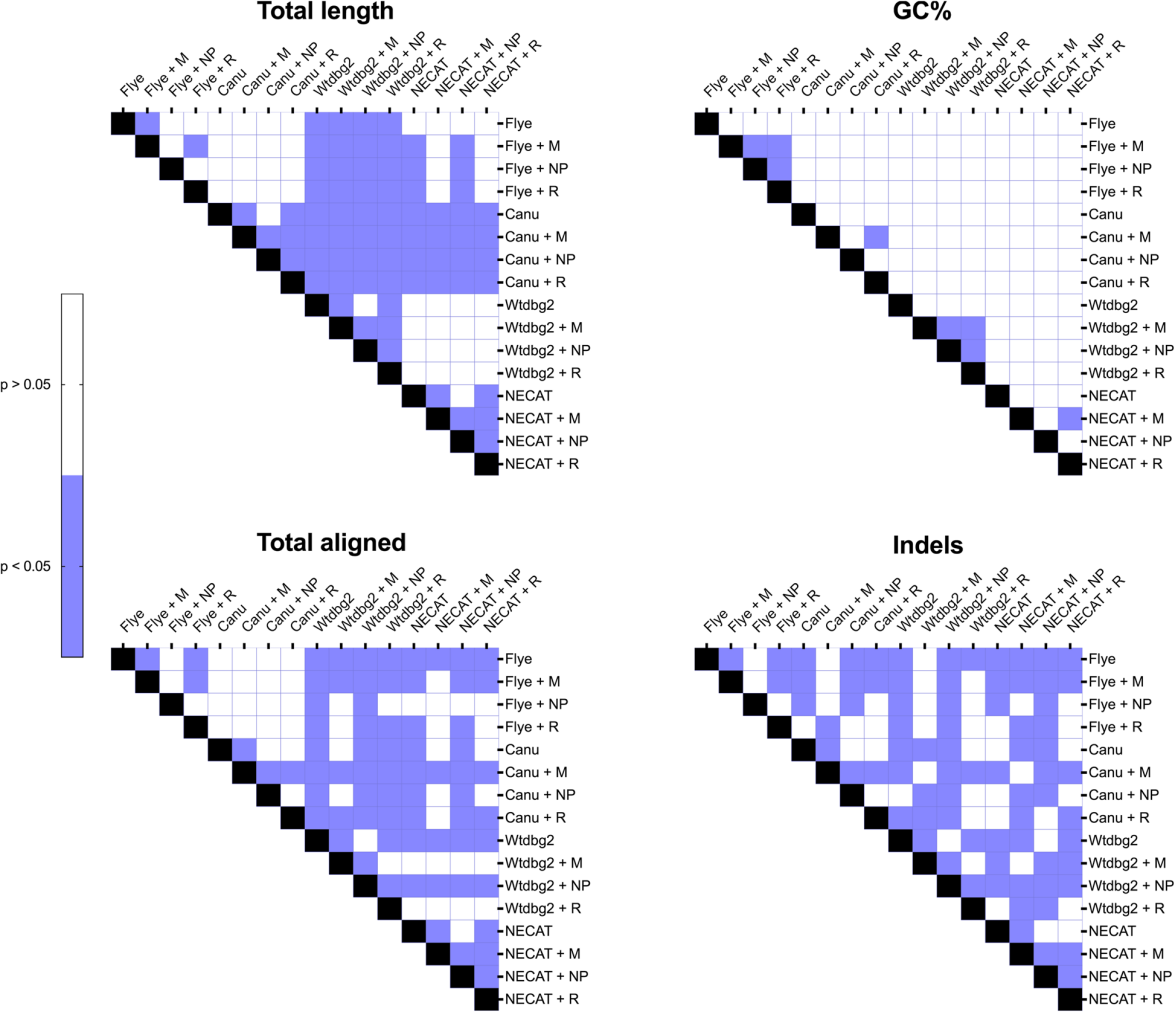
Heatmap statistical analysis for QUAST results. M = Medaka, NP = NextPolish, R = Racon. Quast-based assembly statistics for assembled genomes with and without using read correction tools, including total length (bp), GC%, total aligned (bp), and indels. The Wilcoxon signed-rank test was performed for group comparison

Oxford Nanopore Sequencing technology generates long-reads that overcome NGS limitations, especially when sequencing repeated tandems. However, the performance and optimization of read assembly and read correction tools of ONT long reads still warrant further investigation. In this study, we aimed to evaluate four read assembly (Flye, Canu, Wtdbg2 and NECAT) and three read correction (Medaka, NextPolish and Racon) tools in assembling nine clinical *E. coli* isolates. Since a reference strain was not available for this study, we evaluated assembly accuracy by aligning assemblies to a reference genome obtained from the NCBI database (NC_002655.2, NZ_CP017979.1, NZ_CP051263.1, and NC_007779.1), and therefore, the primary purpose was to assess read assembly and correction tools in gene structure and completeness. To evaluate the effect of different de novo assembly and correction tools on the strain and serotype identification, we used both BLAST + and ECTyper tools. The data presented in Tables 1 and 2 indicate that all assembled genomes belongs to the same strains and serotypes (MLST prediction is presented in Suppl. S1 Table S5). The identified strains from BLAST + were then used as references in QUAST for evaluating assembly and correction tools (Table 3). The four de novo assembly tools could generate consensus files for all nine isolates except for NECAT, which failed to assemble the genomes of two samples (barcodes 02 and 08) due to the high number of short reads and/or the high number of contigs. The assembled genomes’ total length (bp) was higher when using Flye and Canu (Table 3). The larger genome assemblies produced by read assembly tools show the ONT advantage of sequencing organisms with moderate GC content. In general, all corrected assemblies improved total genome length and were significantly (*p* < 0.05) improved when using Medaka and Racon as read correction tools but not NextPolish (Fig. 1). Similarly, Wang et al. have reported significant improvement in genome sizes after using read correction tools [21]. In their study, the reads were corrected by up to 57%. In this study, Wtdbg2 generated the shortest assembled genomes, followed by NECAT (Table 3). The performance of Wtdbg2 and NECAT for assembling ONT reads has been controversial. While several studies have suggested that Wtdbg2 and NECAT perform well in assembling good- and low-quality ONT reads, especially after correcting with Medaka [12, 18, 24], the same was not noticed in this study. The reason could be the methods of DNA extraction used, library preparation and/or the bacteria being sequenced. The number of contigs was not significantly affected by read correction tools and were all the same before and after read corrections (except for barcode 12). Most read correction tools performed well in terms of GC content compared to non-corrected assemblies. Only in barcode 12, the number of GC% was significantly (*p* < 0.05) lower in non-corrected reads. However, Medaka had significantly (*p* < 0.05) lower GC%. The number of indels was significantly (*p* < 0.05) lower when using Flye (with and without using correction tools) and significantly (*p* < 0.05) higher when using Wtdbg2 (Table 3, Fig. 1). The use of Medaka and Racon read correction tools significantly (*p* < 0.05) lowered the number of indels when using Wtdbg2. The usage of read correction tools to lower indels number was also detected by others [38]. The relatively high number of indels is continuously noticed with ONT sequencing, which could introduce errors (such as a stop codon) that affects gene annotation [7, 39].

**Table 1:** Strain identification using ONT long reads with different assembly and read correction tools as predicted by BLAST + tool

**Sample**	**Strain**
Barcode 01	*Escherichia coli* O157:H7 str. EDL933
Barcode 02	*Escherichia coli* str. K-12 substr. W3110
Barcode 03	*Escherichia coli* O157:H7 str. EDL933
Barcode 04	*Escherichia coli* CFT073
Barcode 06	*Escherichia coli* O157:H7 str. EDL933
Barcode 08	*Escherichia coli* str. K-12 substr. W3110
Barcode 09	*Escherichia coli* O157:H7 str. EDL933
Barcode 11	*Escherichia coli* O157:H7 str. EDL933
Barcode 12	*Escherichia coli* O157:H7 str. EDL933

**Table 2:** Serotype identification of clinical *E. coli* isolates using ONT long reads with different assembly and read correction tools as predicted by ECTyper tool

**Sample**	**Serotype**
Barcode 01	O102:H6
Barcode 02	-:H4
Barcode 03	O138:H48
Barcode 04	O81:H27
Barcode 06	O169:H9
Barcode 08	-:H4
Barcode 09	O15:H18
Barcode 11	O8:H12
Barcode 12	O77/O17/O44/O106:H18

**Table 3:** De novo assembly of clinical *E. coli* strains with ONT reads using Flye, Canu, Wtdbg2, and NECAT assemblers with and without read correcting with Medaka, NextPolish, and Racon. M = Medaka, NP = NextPolish, R = Racon. Bold = highest number, underline = lowest number, NA = not applicable

Assembler	Flye					Canu				Wtdbg2				NECAT				
Read correction	-	M	NP	R		-	M	NP	R	-	M	NP	R	-		M	NP	R
Total length (bp)																		
Barcode01	5,469,936	5,478,602	5,539,345	5,475,629		5,579,075	**5,593,923**	5,579,075	5,589,830	4,715,179	4,732,533	4,715,179	4,729,394		5,459,846	5,467,399	5,459,846	5,463,665
Barcode02	5,066,917	**5,080,864**	5,066,917	5,075,921		4,615,621	4,638,920	4,615,621	4,632,707	3,359,552	3,477,009	3,359,552	3,465,773		NA	NA	NA	NA
Barcode03	5,294,465	5,302,241	5,294,465	5,300,744		5,303,012	5,315,872	5,303,012	5,314,629	5,213,454	5,238,851	5,213,454	5,237,108		5,314,925	**5,320,631**	5,314,925	5,318,958
Barcode04	5,247,697	5,256,000	5,247,697	5,253,316		5,369,978	**5,385,202**	5,369,978	5,381,812	4,220,087	4,244,567	4,220,087	4,236,146		5,208,954	5,215,762	5,208,954	5,212,343
Barcode06	5,476,326	5,484,941	5,476,326	5,483,418		5,530,456	**5,545,123**	5,530,456	5,542,970	5,410,478	5,437,044	5,410,478	5,434,446		5,475,891	5,482,890	5,475,891	5,481,005
Barcode08	9,671,007	**9,682,127**	9,671,007	9,654,206		5,988,291	6,026,955	5,988,291	6,010,588	4,369,074	4,529,659	4,369,074	4,508,127		NA	NA	NA	NA
Barcode09	7,253,462	7,269,121	7,253,462	7,244,915		7,482,615	**7,521,371**	7,482,615	7,504,387	6,279,759	6,386,039	6,279,759	6,354,810		6,253,465	6,278,638	6,253,465	6,254,786
Barcode11	4,892,227	4,899,205	4,892,227	4,899,245		4,977,509	4,988,711	4,977,509	4,988,061	5,046,387	**5,073,001**	5,046,387	5,050,697		4,648,910	4,653,507	4,648,910	4,653,170
Barcode12	5,515,594	5,520,560	5,511,890	5,517,067		5,709,957	**5,725,656**	5,709,957	5,721,016	5,329,936	5,365,151	5,329,936	5,359,875		5,409,945	5,417,213	5,409,945	5,412,674
Number of contigs																		
Barcode01	5	5	5	5	**7**		**7**	**7**	**7**	**7**	**7**	**7**	**7**		3	3	3	3
Barcode02	126	128	126	128	**207**		**207**	**207**	**207**	127	127	127	127		NA	NA	NA	NA
Barcode03	6	6	6	6	3		3	3	3	**9**	**9**	**9**	**9**		8	8	8	8
Barcode04	13	13	13	13	12		12	12	12	**14**	**14**	**14**	13		13	13	13	13
Barcode06	11	11	11	11	6		6	6	6	**17**	**17**	**17**	**17**		6	6	6	6
Barcode08	357	358	357	356	**537**		**539**	**537**	**537**	209	209	209	209		NA	NA	NA	NA
Barcode09	**137**	**137**	**137**	**137**	127		127	127	127	79	79	79	78		108	108	108	108
Barcode11	1	1	1	1	10		10	10	10	**12**	**12**	**12**	9		1	1	1	1
Barcode12	**18**	**16**	**16**	**16**	15		15	15	15	11	11	11	11		20	20	20	20
GC%																		
Barcode01	50.74	50.74	**50.78**	50.73	50.72		50.72	50.72	50.71	50.74	50.73	50.74	50.71		50.75	50.76	50.75	50.75
Barcode02	50.92	50.92	50.92	50.89	50.84		50.93	50.84	50.88	**51.5**	51.22	**51.5**	51.1		NA	NA	NA	NA
Barcode03	50.8	50.79	50.8	50.79	**50.82**		50.81	**50.82**	50.81	50.81	50.78	50.81	50.77		50.8	50.8	50.8	50.8
Barcode04	50.66	50.65	50.66	50.64	**50.75**		**50.75**	**50.75**	50.74	50.49	50.46	50.49	50.43		50.64	50.65	50.64	50.63
Barcode06	**50.57**	50.56	**50.57**	50.56	50.48		50.47	50.48	50.46	50.52	50.51	50.52	50.49		50.5	50.5	50.5	50.49
Barcode08	52.9	52.88	52.9	52.79	53.15		53.15	53.15	53.03	**54.61**	53.96	**54.61**	53.46		NA	NA	NA	NA
Barcode09	50.21	50.13	50.21	50.06	50.68		50.68	50.68	50.62	50.49	50.41	50.49	50.28		**50.71**	50.7	**50.71**	50.66
Barcode11	50.85	50.84	50.85	50.84	50.77		50.76	50.77	50.76	50.75	50.72	50.75	50.7		**50.95**	**50.95**	**50.95**	50.94
Barcode12	22	50.39	50.4	50.38	22		50.38	50.39	50.37	11	50.44	**50.47**	50.41		18	50.36	50.35	50.34
Total aligned (bp)																		
Barcode01	**4,153,701**	4,131,518	2,489,913	4,088,076	4,089,970		4,149,790	4,089,969	4,098,541	3,435,260	3,582,022	3,435,144	3,513,526		4,072,587	4,137,015	4,072,586	4,073,699
Barcode02	**4,234,234**	4,223,032	4,234,231	4,149,381	3,614,373		3,805,118	3,614,371	3,730,664	1,115,774	2,732,632	1,115,774	2,757,655		NA	NA	NA	NA
Barcode03	**4,450,464**	4,430,100	**4,450,464**	4,410,942	4,405,608		4,441,610	4,405,608	4,426,312	4,140,253	4,385,142	4,140,253	4,370,266		4,385,814	4,443,930	4,385,814	4,415,394
Barcode04	**4,223,485**	4,182,278	**4,223,485**	4,163,481	4,152,322		4,204,149	4,152,322	4,165,641	3,217,785	3,470,346	3,217,785	3,432,050		4,096,702	4,189,276	4,096,702	4,153,810
Barcode06	**4,430,645**	4,406,775	**4,430,645**	4,376,047	4,365,538		4,409,083	4,365,339	4,372,342	4,147,601	4,375,901	4,147,601	4,336,730		4,289,285	4,396,616	4,289,128	4,355,667
Barcode08	3,949,545	3,951,123	3,949,544	**3,955,591**	2,610,245		2,669,926	2,610,245	2,667,499	335,832	1,185,183	335,832	1,391,558		NA	NA	NA	NA
Barcode09	4,679,828	4,626,706	4,679,828	4,590,052	5,601,691		**5,633,641**	5,601,691	5,502,302	3,426,173	4,258,633	3,426,173	4,304,669		4,593,133	4,669,739	4,593,133	4,621,159
Barcode11	4,126,820	4,107,981	**4,126,846**	4,082,815	4,074,616		4,104,945	4,074,642	4,069,038	3,915,825	4,109,486	3,915,851	4,067,450		3,873,085	3,909,040	3,873,111	3,873,759
Barcode12	**4,110,855**	4,086,413	**4,110,855**	4,065,379	4,057,280		4,105,650	4,057,280	4,081,821	3,656,460	4,072,434	3,656,453	4,025,549		3,966,073	4,045,206	3,965,670	4,009,086
Indels																		
Barcode01	3369	4497	**9003**	5765	6088		4475	6088	6026	8020	4006	8019	5368		6899	4601	6899	5946
Barcode02	3363	4484	3363	6801	11,882		4379	11,882	7155	**12,724**	7131	**12,724**	6097		NA	NA	NA	NA
Barcode03	3094	4423	3094	5826	6265		4379	6265	6080	**13,714**	4438	**13,714**	6120		8115	4581	8115	6020
Barcode04	3031	4129	3031	5484	6350		4004	6350	5771	**11,303**	3634	**11,303**	5024		8076	4348	8076	5687
Barcode06	3197	4403	3197	6135	6346		4327	6346	6290	**12,523**	4587	**12,523**	6527		8256	4613	8254	6251
Barcode08	7221	7447	7221	**9096**	8513		4225	8513	6916	4077	4412	4077	4924		NA	NA	NA	NA
Barcode09	7054	8617	7054	10,217	13,117		10,696	13,117	14,279	**24,432**	11,822	24,431	10,949		11,868	8790	11,868	11,192
Barcode11	2867	3990	2867	5678	5206		3837	5206	5731	**9919**	4053	**9919**	5821		6519	3942	6519	5394
Barcode12	3232	4313	3253	5451	3648.64		4338	6042	5714	3537.49	4819	15,282	5811		**3616.25**	4626	7715	5691

**Fig. 2:**
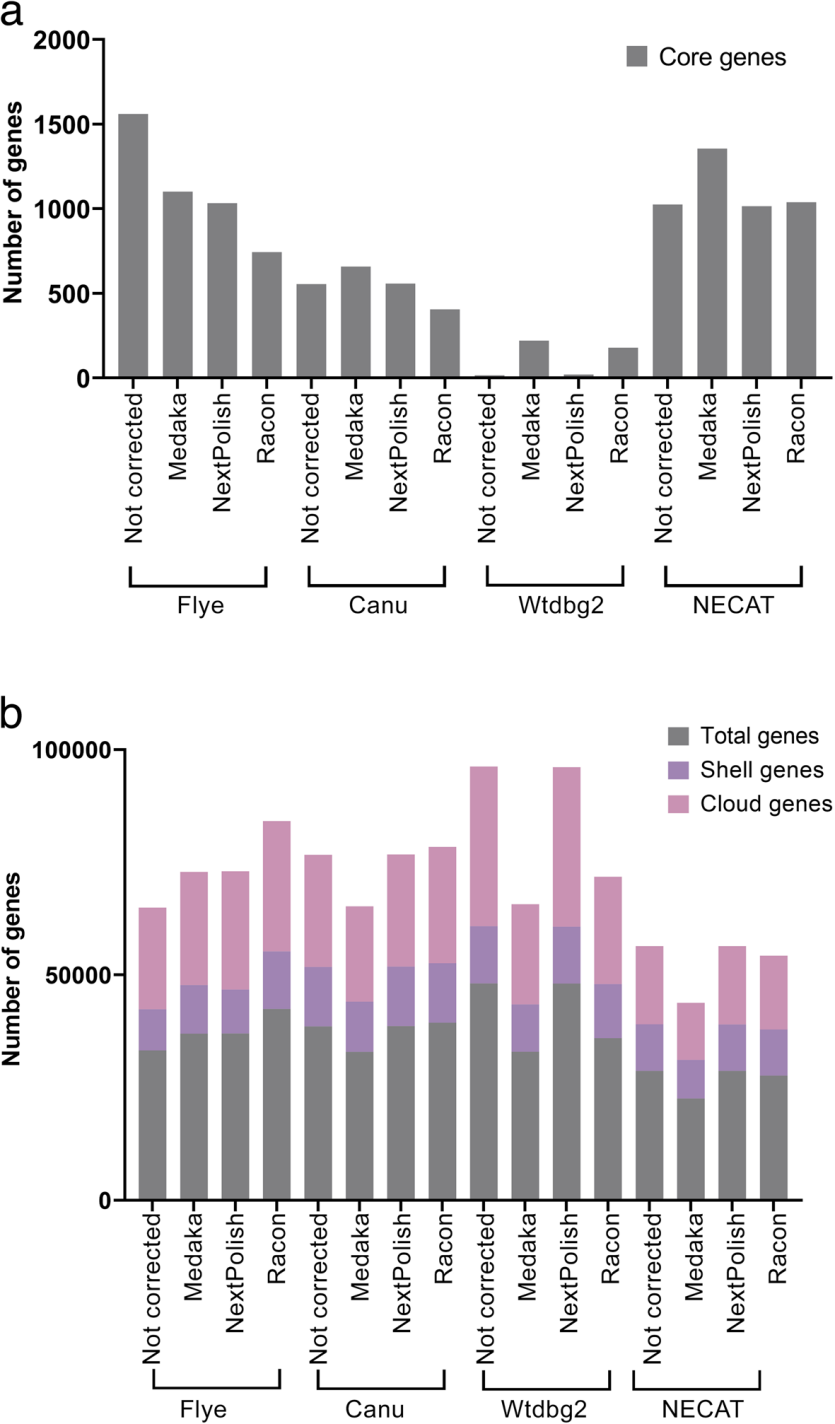
Pan-genomes of nine clinical *E. coli* isolates using Flye, Canu, Wtdbg2 and NECAT as read assemblers with and without Medaka, NextPolish and Racon read correction tools. **a** The total number of core genes present in all isolates, **b** The total number of accessory (shell and cloud) genes. Both of the core and the accessory genes were perfomed using the annotation tool (Prokka) and pan-genome tool (Roary)

A total of 15 assembled genomes (12 corrected and three non-corrected) per sample were included in the pan-genome analysis, which displayed core and accessory (shell and cloud) genes. Flye was the most effective assembler for the pan-genome analysis, followed by NECAT and Canu (Fig. 2a and b). Non-corrected Flye assemblies consisted of total genes of 33,257, 1,560 (4.6%) of which were core genes and 31,697 (95%) were accessory genes. The total number of indels was the lowest in genomes assembled by Flye. Since the total number of indels detected by read assembly and correction tools affects gene annotation in which the high read errors produce more misannotated genes and the number of accessory genes, this could explain the good performance of Flye in detecting the highest core genes [38]. Interestingly, genomes assembled by Canu, Wtdbg2, and NECAT had more core genes when corrected by Medaka compared to non-corrected assemblies (Fig. 2a). Although Wtdbg2 showed the highest number of total genes 48,132, it showed the least number of core genes (16 – 0.03%). This could be due to the inaccurate genome size produced by Wtdbg2 and a high number of indels detected. Genomes assembled by NECAT (with and without correction) had the lowest number of total and accessory genes (Fig. 2b). The use of Racon as a read correction tool for genomes assembled by Flye and Canu increased the number of total and accessory genes (Fig. 2b). However, this was not noticed in genomes assembled by Wtdbg2 and NECAT.

**Fig. 3:**
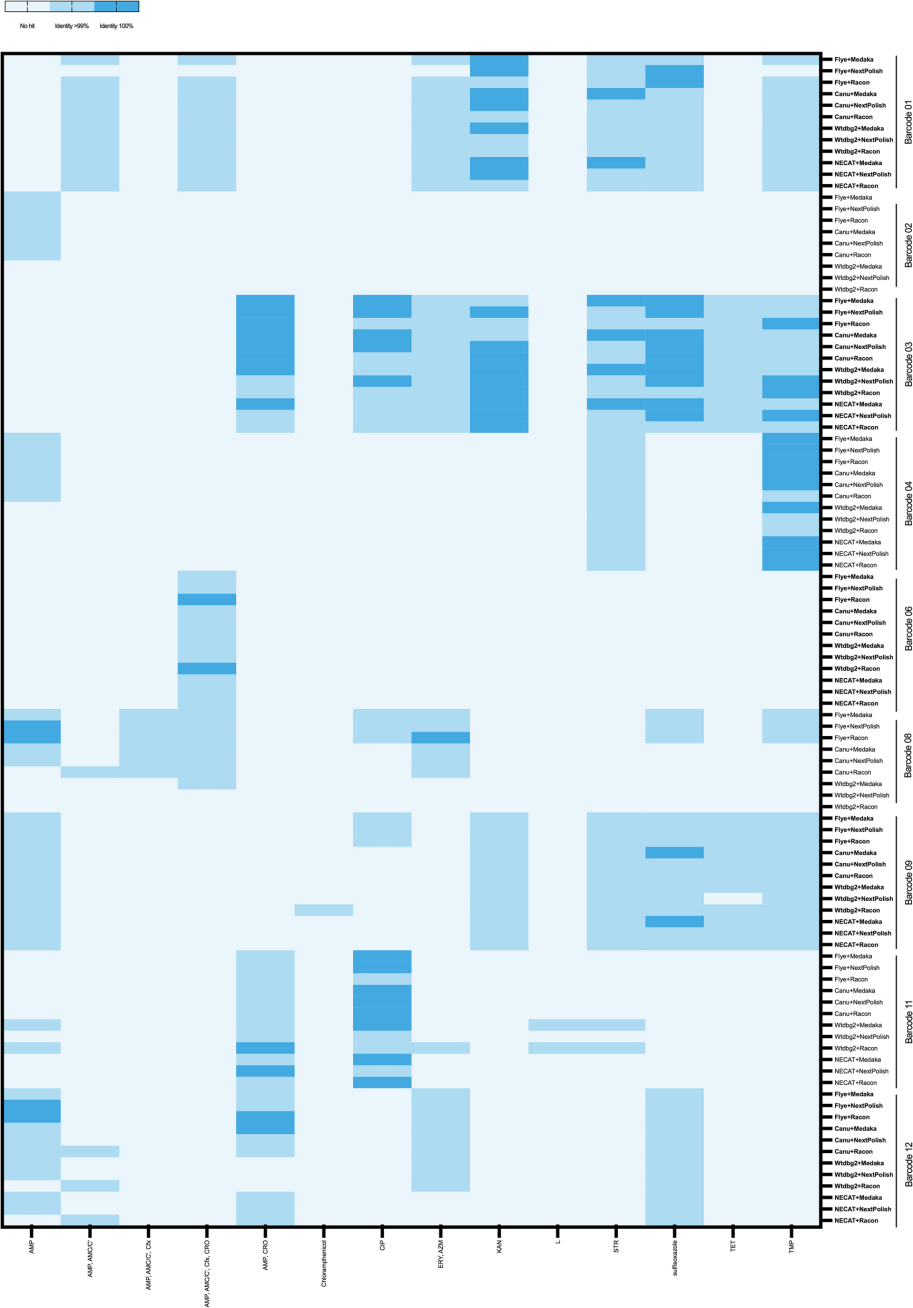
Heatmap presenting antimicrobial resistance identification by staramr (ResFinder) of nine clinical *E. coli* isolates using Flye, Canu, Wtdbg2 and NECAT as read assemblers with Medaka, NextPolish and Racon read correction tools. AMP = ampicillin, AMC/C’ = amoxicillin/clavulanic acid, Cfx = cefoxitin, CRO = ceftriaxone, CIP = ciprofloxacin, ERY = erythromycin, AZM = azithromycin, KAN = kanamycin, L = lincomycin, STR = streptomycin, TET = tetracyclin, TMP = trimethoprim. Staramr classified the presence of the resistance genes to 100% identity, > 99% identity, and no hits based to the corresponding colors

**Fig. 4:**
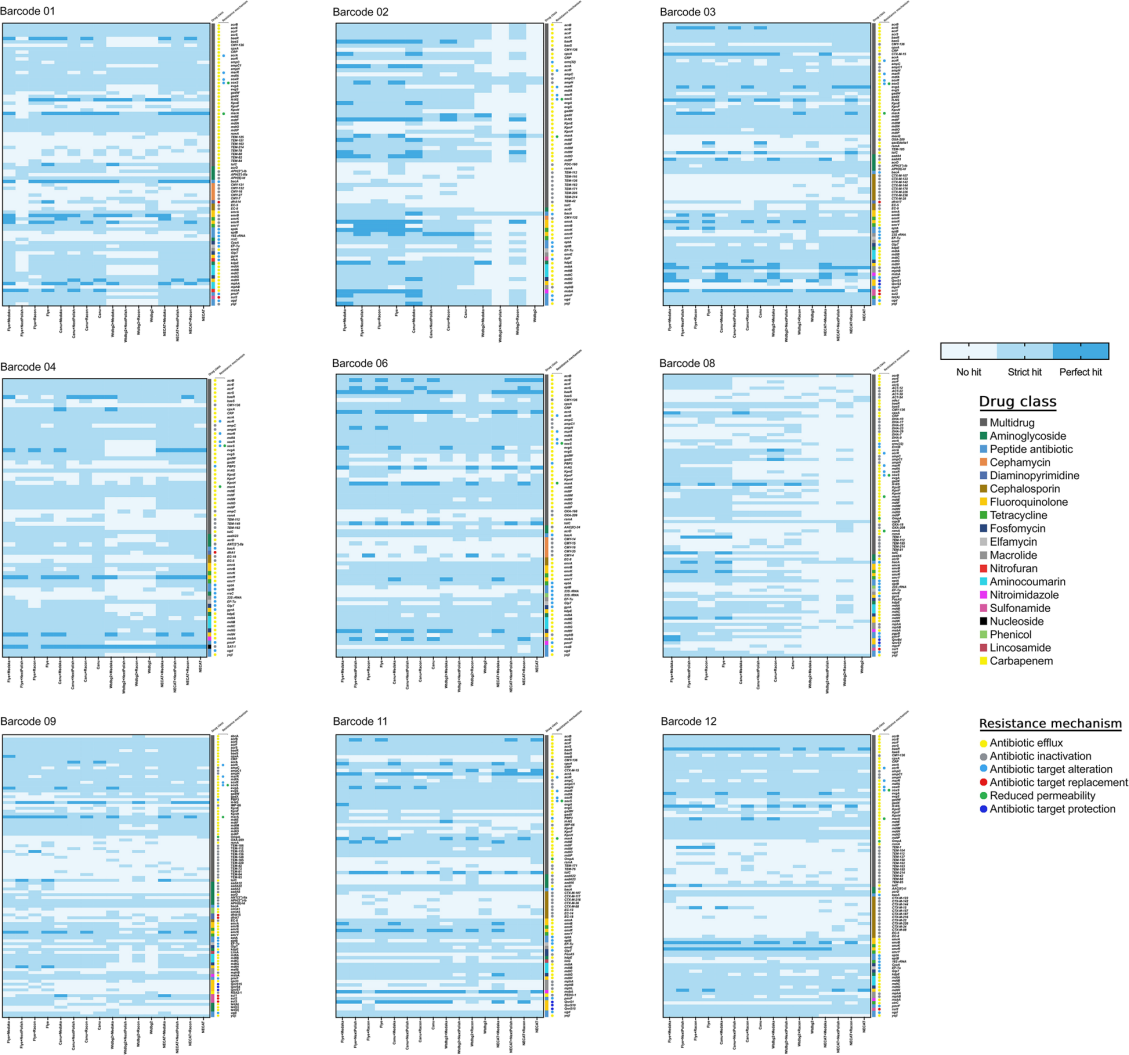
Complex heatmaps of antimicrobial resistance gene class and mechanism identification by RGI of nine clinical *E. coli* isolates using Flye, Canu, Wtdbg2 and NECAT as read assemblers with Medaka, NextPolish and Racon read correction tools. Each isolate represented in a separate heatmap with predicted resistence genes, the drug class and the drug mechanism. The RGI predicted the perfomence of 19 drug classes and 6 drug mechanisms given that the RGI classified the precence of the resistance genes to perfect hit, strict hit and no hit based to the corresponding colors

A major advantage of ONT sequencing is the rapid identification of antimicrobial resistance genes, plasmids, and virulence genes in bacterial genomes [40, 41]. Besides, the long-reads generated allow the detection of the presence/absence of antimicrobial and virulence genes and their architectures i.e., chromosomal vs. plasmid [42]. In this study, we investigated nine clinical *E. coli* isolates. Antimicrobial resistance genes were detected using two independent tools: staramr (ResFinder) and RGI, the plasmid detection by PlasmidFinder, and virulence genes by Abricate. The results obtained from these tools did not follow a particular pattern, however, some read and correction tools performed better than others. Flye, Canu and NECAT read assembly tools performed well in detecting antimicrobial resistance genes when using staramr with Flye being the best assembler to identify resistant genes in genomes regardless of the correction tool used followed by Canu (Fig. 3). Both Flye and Canu were able to identify resistance genes by 98–99% or 100% identity while Wtdbg2 and NECAT missed these genes (barcodes 02, 04, 08, 09, and 12). Interestingly, only Wtdbg2/Racon was able to detect resistance in chloramphenicol in barcode 09, and ampicillin (AMP), erythromycin (ERY), lincomycin (L) and streptomycin (STR) in barcode 11. The RGI analysis followed staramr results to a certain degree. For example, Wtdbg2 did not detect the presence of *baeR*, *baeS*, and *CMY-136* genes -antibiotic efflux genes that confer resistance to multiple drug classes- in barcode 01, and most resistance genes in barcode 02 (Fig. 4). However, this was improved when using Medaka and Racon read correct tools (Fig. 4). Although hybrid assembly between short- and long-reads is recommended for more accurate antimicrobial resistance profiling [43], we have noticed that usage of Medaka and Racon read correction tools enhanced Wtdgb2 prediction in which 133 genes conferring resistance to different antibiotic classes were detected across all samples (Fig. 4).

**Fig. 5:**
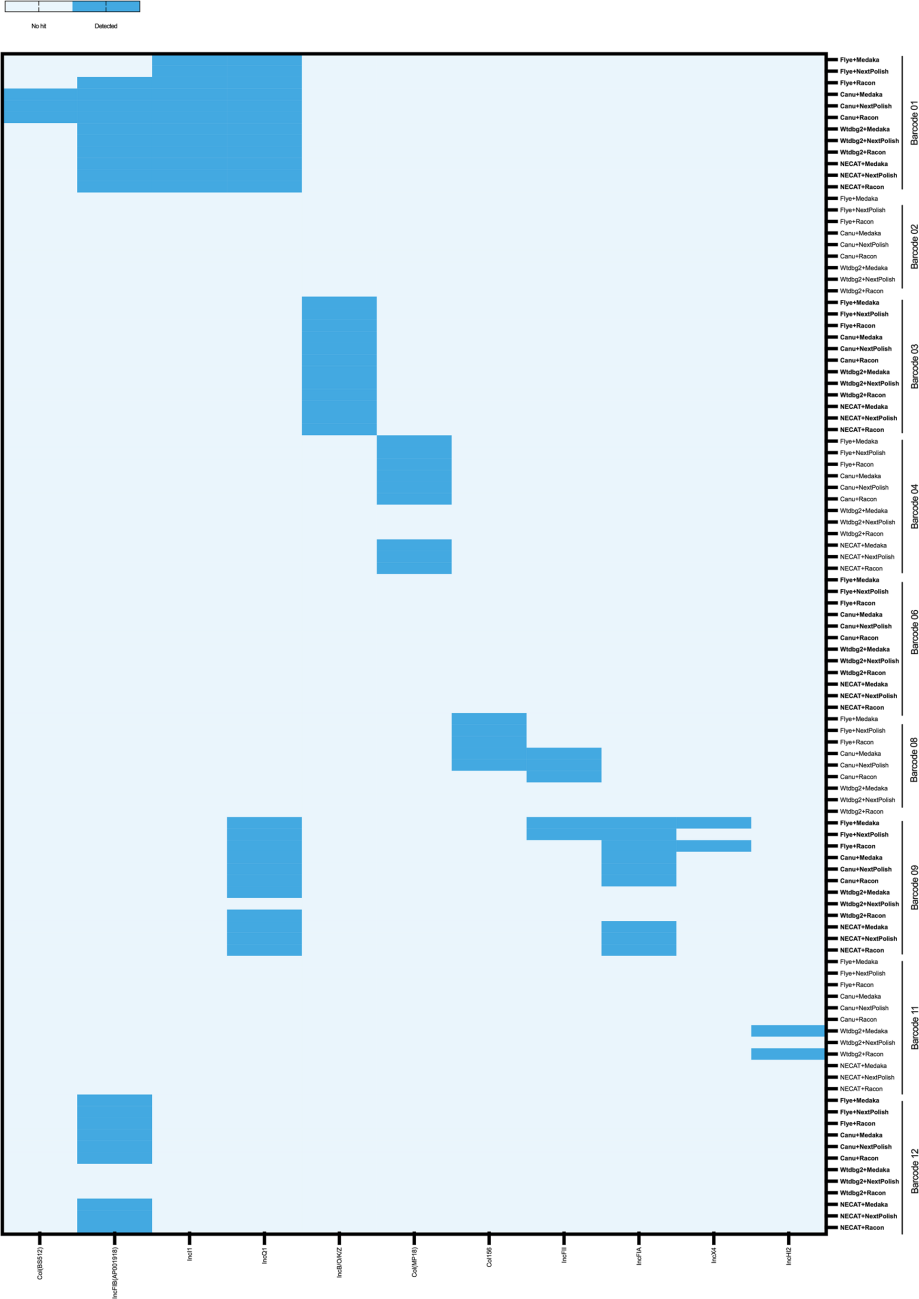
Plasmid identification by staramr (PlasmidFinder) of clinical *E. coli* using Flye, Canu, Wtdbg2 and NECAT as read assemblers with Medaka, NextPolish and Racon read correction tools

The plasmid detection results using PlasmidFinder were also inconsistent. Generally, Canu performed the best predicting most of the plasmids in all samples, however, it failed to predict the presence of IncX4 plasmid in barcode 09 and IncHI2 plasmid in barcode 11, which was only detected by Flye and Wtdgb2 after correction with Medaka and Racon but not NextPolish. Surprisingly, Wtdbg2 (with and without correction) could not detect the presence of all Col plasmids (ColBS512, ColMP18, and Col156) as well as IncFI1 and IncFII, while Flye failed to detect ColBS156, IncFI1, and IncHI1. In this study, we detected antimicrobial resistance genes detection on plasmids in four isolates (Table 4). For example, In barcode 01 several resistance genes *blaCMY*-7, *aph(6*), *blaTEM-34*, *dfrA14*, *mph(A)*, *strA*, and *sul2* were detected on two plasmids, IncI1 And IncQ1. Not all read assembly and correction tools were able to detect the presence of these genes on plasmids. Reads assembled by Flye and corrected by NextPolish could not detect *blaTEM*, *dfreA*, and *mph*. In barcode 03, reads assembled by Wtdbg2 could not detect the *sul2* gene when using staramr (ResFinder), however, the gene was detected when using RGI (Figs. 4 and 5). Contradictory, the same gene was detected in barcode 09 by reads assembled by Canu and corrected by NextPolish when using staramr (ResFinder) but not RGI. Although long reads have shown better detection of plasmids than short reads [44], the inconsistency of plasmid detection when using multiple read and correction tools was also noted in other studies [11, 38]. George et al. reported that regardless of the read assembly tool used for long reads, some small-sized plasmids were missed and only retained when using hybrid assembly [7]. This means that library preparations, bioinformatic tools, and/or sequencing technology for long-reads still need to be improved for accurate plasmid detection.

**Table 4:** Antimicrobial resistance gene on plasmids predicted by ResFinder and PlasmidFinder after de novo assembly of clinical E. coli strains with ONT reads using Flye, Canu, Wtdbg2, and NECAT assemblers with and without read correcting with Medaka, NextPolish, and Racon. F = Flye, C = Canu, W = Wtdbg2, NE = NECAT, M = Medaka, NP = NextPolish, R = Racon

Sample	Plasmid	Gene	Predicted phenotype	Detected by
Barcode 01	IncI1	*blaCMY-7*	Ampicillin, Amoxicillin/clavulanic acid, Cefoxitin, ceftriaxone	F + M, F + R, C + M, C + NP, C + R, W + M, W + NP, W + R
	IncQ1	*aph(6)-Id*	Kanamycin	All
		*blaTEM-34*	Ampicillin, Amoxicillin/clavulanic acid	All except F + NP
		*dfrA14*	Trimethoprim	All except F + NP
		*mph(A)*	Erythromycin, azithromycin	All except F + NP
		*strA*	Streptomycin	All
		*sul2*	Sulfisoxazole	All
Barcode 03	IncB/O/K/Z	*aadA5*	Streptomycin	All except NE + NP
		*aph(6)-Id*	Kanamycin	All
		*blacTX-M-15*	Ampicillin, ceftriaxone	All
		*dfrA17*	Trimethoprim	All
		*mph(A)*	Erythromycin, azithromycin	All
		*qnrS1*	Ciprofloxacin I/R	All
		*strA*	Streptomycin	All
		*sul1*	Sulfisoxazole	All
		*sul2*	Sulfisoxazole	All except W + M, W + NP, W + R
		*tet(A)*	Tetracycline	All
Barcode 08	IncFII	*blaTEM-S7*	Ampicillin	C + M, C + NP
		*mph(A)*	Erythromycin, azithromycin	C + M, C + NP, C + R
		*blaTEM-79*	Ampicillin	C + R
Barcode 09	InCQ1	*aph(3'')-Ib*	Streptomycin	All
		*aph(6)-Id*	Kanamycin	All
		*blaTEM-1B*	Ampicillin	All except F + M, F + NP, F + R
		*dfrA7*	Trimethoprim	All except F + M, F + NP, F + R
		*sul1*	Sulfisoxazole	All except F + M, F + NP, F + R
		*sul2*	Sulfisoxazole	All except W + NP
		*tet(A)*	Tetracycline	NE + M, NE + NP, NE + R

**Fig. 6:**
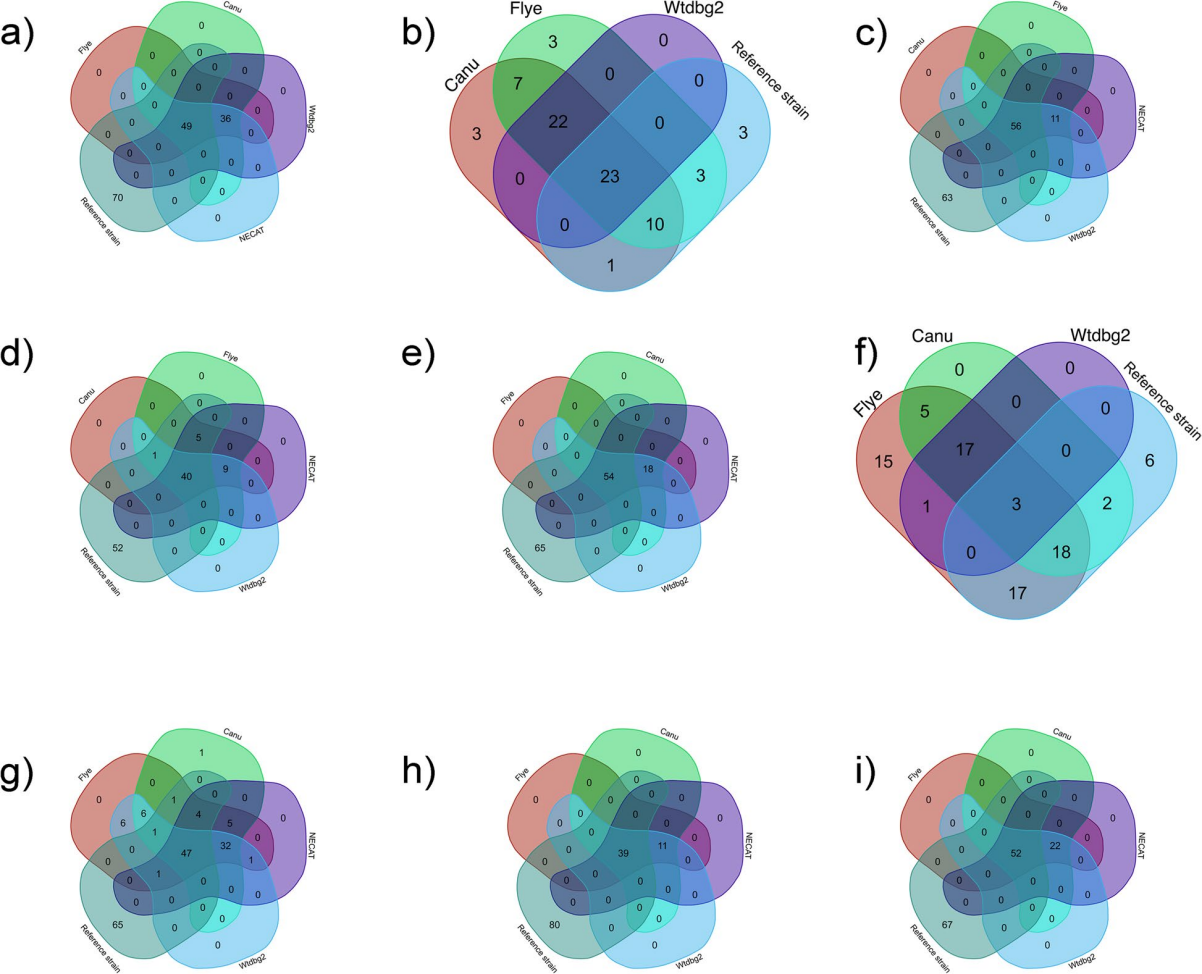
Venn-diagram of virulence factors gene identification by ABRicate of nine clinical *E. coli* isolates using Flye, Canu, Wtdbg2 and NECAT as read assemblers. The reference genome for each isolate was used based on BLAST + results shown in Table 1. The diagram shows the number of the virulence factors overlaped between the reference strain and the three genome assembly tools. **a** barcode 01, **b** barcode 02, **c** barcode 03, **d** barcode 04, **e** barcode 06, **f** barcode 08, **g** barcode 09, **h** barcode 11, and **i** barcode 12

Identifying virulence factors is critical in understanding *E. coli* pathogenicity that may impact human health [40]. In this study, we did not notice a significant difference in virulence factors detected after using different read correction tools when using ABRicate (Suppl. S2). Among the nine clinical *E. coli* isolates differences in virulence detection were noticed in four samples (barcodes 02, 04, 08, and 09) which followed a particular pattern (Fig. 6). Flye performed as the best read assembly tool, followed by Canu, Wtdbg2, and then NECAT. Flye was also able to detect virulence genes on the plasmids. In barcode 08 the gene encoding for enterotoxin *senB* was detected on Col156 plasmid. Besides, Flye, Canu, and NECAT were able to detect the *iroB*, *iroC*, *iroD*, *iroE*, *iroN*, genes on the IncFIA plasmid in barcode 09. Although a reference strain was included in this analysis, the clinical strains may not necessarily match the total number of virulence factors detected in the reference strain. A shortcoming of this study is the unavailability of another sequencing method as a reference. Therefore, we could not be definitive regarding the number of virulence factors nor if any gene was lost during library preparation. The usage of long reads is arguably better for the rapid detection of virulence factors. Obtaining a circular/closed genome with fewer read errors is much more robust in outbreak surveillance and investigations [40, 45, 46]. Unfortunately, although improved, different virulence factors were detected when applying multiple bioinformatic tools [47].

Third-generation sequencing tools, such as ONT, are acceptable options for whole-genome sequencing especially in low to mid income countries due to their affordability and simplicity. The relatively higher per-read error rate of ONT, which necessitates different assembly and correction approaches to transform raw signals into completely assembled genomes can be reduced by using freely available read assembly and read correction tools. There is necessity of benchmarking real data sets from clinical isolates. In this study, we found that the use of mix-and-matched read assembly and read correction tools can lead to significant differences in total bacterial length, AMR detection, and plasmid and virulence factor identification.

## Supplementary Information

**Additional file 1:** **Table S1.** Accession numbers of read sets. **Table S2.** Overview of the sequencing run. **Table S3.** Nanoplot statistics before and after filtering. **Table S4.** Benchmark the running time and CPU usage of read trimming, assembly, read correction, quality control and tertiary analysis tools. **Table S5.** MLST prediction by staramr.

**Additional file 2.** 

## Data Availability

The sequencing reads were submitted to EMBL's European Bioinformatics Institute and are available online with accession numbers ERR10468513- ERR10468521 (Suppl. S1 Table S1) available at: https://www.ebi.ac.uk/ena/browser/view/PRJEB57325.

## Abbreviations

AMC: Amoxicillin
AMP: Ampicillin
AZM: Azithromycin
bp: Base pair
Cfx: Cefoxitin
CIP: Ciprofloxacin
CRO: Ceftriaxone
DNA: Deoxyribonucleic acid
ERY: Erythromycin
KAN: Kanamycin
MGE: Mobile genetic element
NGS: Next generation sequencing
OLC: Over-layout consensus
ONT: Oxford Nanopore Technology
RGI: Resistance gene identifier
RNA: Ribonucleic acid
SNP: Single nucleotide polymorphism
STR: Streptomycin
TET: Tetracycline
TMP: Trimethoprim
