# Supplementary material for: The impact of applying various de novo assembly and correction tools on the identification of genome characterization, drug resistance, and virulence factors of clinical isolates using ONT sequencing
Source: BMC Biotechnol. 2023 Jul 31;23:26. doi: 10.1186/s12896-023-00797-3 (PMC10391896; doi:10.1186/s12896-023-00797-3)
Supplement: Supplementary file 1 — Additional file 1: Table S1. Accession numbers of read sets. Table S2. Overview of the sequencing run. Table S3. Nanoplot statistics before and after filtering. Table S4. Benchmark the running time and CPU usage of read trimming, assembly, read correction, quality control and tertiary analysis tools. Table S5. MLST prediction by staramr. [file 12896_2023_797_MOESM1_ESM.docx]

Table S1. Accession numbers of read sets

| Sample | Accession number | File name |
| --- | --- | --- |
| Barcode 01 | ERR10468513 | barcode01all.fastq.gz |
| Barcode 02 | ERR10468514 | barcode02all.fastq.gz |
| Barcode 03 | ERR10468515 | barcode03all.fastq.gz |
| Barcode 04 | ERR10468516 | barcode04all.fastq.gz |
| Barcode 06 | ERR10468517 | barcode06all.fastq.gz |
| Barcode 08 | ERR10468518 | barcode08all.fastq.gz |
| Barcode 09 | ERR10468519 | barcode09all.fastq.gz |
| Barcode 11 | ERR10468520 | barcode11all.fastq.gz |
| Barcode 12 | ERR10468521 | barcode12all.fastq.gz |

Table S2. Overview of the sequencing run.

| Barcode | gDNA concentration (ng/µl) | gDNA purity (260/280) | Input mass (ng) | After end prep (ng/µl) | After barcoding (ng/µl) | Volume to pool (µl) | Pooled samples (ng/µl) | Pooled samples after adapter ligation (ng/µl) |
| --- | --- | --- | --- | --- | --- | --- | --- | --- |
| 1 | 12.4 | 1.87 | 1000 | 70.8 | 10.3 | 5.5 | 11.8 | 29.8 |
| 2 | 21.6 | 1.56 |  | 43.6 | 17.3 | 3.2 |  |  |
| 3 | 12 | 1.87 |  | 72 | 12.5 | 4.5 |  |  |
| 4 | 5 | 1.85 |  | 65 | 16.6 | 3 |  |  |
| 6 | 41.1 | 1.83 |  | 43.8 | 13.3 | 4 |  |  |
| 8 | 18.3 | 1.68 |  | 40.8 | 18.9 | 3 |  |  |
| 9 | 16.6 | 1.89 |  | Too high* | 24.1 | 2.3 |  |  |
| 11 | 4.97 | 1.94 |  | 61.6 | 12.8 | 4.3 |  |  |
| 12 | 38.9 | 1.73 |  | 39.6 | 14 | 4 |  |  |

* Too high = out of kit detection range 0.005-120 ng/ul (more than 120 ng/µl)

Table S3. Nanoplot statistics before and after filtering

| Sample | Mean read length | | Mean read quality | | Number of reads | | Number of bases above >Q8 | |
| --- | --- | --- | --- | --- | --- | --- | --- | --- |
|  | Before | After | Before | After | Before | After | Before | After |
| Barcode 01 | 3,709 | 2,824.1 | 11.1 | 11.1 | 58,837 | 57,094 | 58,837 | 57,094 |
| Barcode 02 | 628 | 555.8 | 11.1 | 11.1 | 444,593 | 439,458 | 444,593 | 439,458 |
| Barcode 03 | 1,149 | 871 | 11.2 | 11.2 | 265,086 | 257,307 | 265,086 | 257,307 |
| Barcode 04 | 1,071.8 | 800.6 | 11.2 | 11.2 | 255,170 | 249,157 | 255,170 | 249,157 |
| Barcode 06 | 1,102.8 | 837.8 | 11.3 | 11.3 | 307,353 | 301,456 | 307,353 | 301,456 |
| Barcode 08 | 1,133.3 | 948.5 | 11.2 | 11.2 | 124,195 | 122,008 | 124,195 | 122,008 |
| Barcode 09 | 1,849.4 | 1,312.7 | 11.2 | 11.2 | 76,365 | 74,374 | 76,365 | 74,374 |
| Barcode 11 | 1,735.8 | 1,382.1 | 11.2 | 11.2 | 179,818 | 174,471 | 179,818 | 174,471 |
| Barcode 12 | 1,939.7 | 1,603.1 | 11.2 | 11.2 | 114,622 | 111,357 | 114,622 | 111,357 |

Table S4. Benchmark the running time and CPU usage of read trimming, assembly, read correction, quality control and tertiary analysis tools

| Tool |  | CPU* | Time (seconds) |
| --- | --- | --- | --- |
| Sequencing reads trimming | Porechop | 16 | 564 |
| Assembly | Flye | 16 | 347 |
|  | NECAT | 12 | 504 |
|  | Canu | 16 | 2883 |
|  | Wtdbg2 | 16 | 2 |
| Read correction | Medaka | 5 | 1,942 |
|  | NextPolish | 2 | 26 |
|  | Racon | 11 | 346 |
| Quality control | Quast | 16 | 5 |
|  | Nanoplot | 15 | 3 |
| Tertiary analysis | Roary | 16 | 47 |
|  | Prokka | 8 | 183 |
|  | StarAMR | 4 | 3 |
|  | RGI | 12 | 90 |
|  | Abricate | 2 | 2 |

*CPU = The Central Processing Unit

Table S5. MLST prediction by staramr

| Barcode | Scheme | Sequence type | Locus 1 | Locus 2 | Locus 3 | Locus 4 | Locus 5 | Locus 6 | Locus 7 |
| --- | --- | --- | --- | --- | --- | --- | --- | --- | --- |
| 01 | ecoli | 7395 | *adk*(35) | *fumC*(37) | *gyrB*(29) | *icd*(25) | *mdh*(4) | *purA*(564) | *recA*(73) |
| 02 | ecoli | 5507 | *adk*(10) | *fumC*(11) | *gyrB*(4) | *icd*(560) | *mdh*(8) | *purA*(8) | *recA*(2) |
| 03 | ecoli | 219 | *adk*(58) | *fumC*(53) | *gyrB*(53) | *icd*(58) | *mdh*(24) | *purA*(1) | *recA*(42) |
| 04 | ecoli | 452 | *adk*(76) | *fumC*(43) | *gyrB*(19) | *icd*(37) | *mdh*(30) | *purA*(1) | *recA*(25) |
| 06 | ecoli | N/A | *adk*(621?) | *fumC*(136) | *gyrB*(110) | *icd*(878?) | *mdh*(80) | *purA*(1) | *recA*(2) |
| 08 | ecoli | N/A | *adk*(~773) | *fumC*(11) | *gyrB*(4) | *icd*(389?) | *mdh*(8) | *purA*(8) | *recA*(33?) |
| 09 | ecoli | N/A | *adk*(225?) | *fumC*(1133?) | *gyrB*(110) | *icd*(~878) | *mdh*(80) | *purA*(~404) | *recA*(11) |
| 11 | ecoli | N/A | *adk*(6) | *fumC*(65) | *gyrB*(344) | *icd*(~560) | *mdh*(513?) | *purA*(13) | *recA*(6) |
| 12 | ecoli | N/A | *adk*(21) | *fumC*(35) | *gyrB*(61?) | *icd*(52) | *mdh*(5) | *purA*(565?) | *recA*(4) |

N/A: Not applicable
